# Study on the relationship between insomnia disorder, PET/CT, and gut microbiota in patients with Alzheimer’s disease

**DOI:** 10.3389/fneur.2025.1669835

**Published:** 2025-11-13

**Authors:** Jian Kang, Jianwen Chen, Peixue Li, Shiqi Zhao, Fangfang Jing, Jinghui Xie, Chunbo Dong

**Affiliations:** 1 Department of Neurology, The First Affiliated Hospital of Dalian Medical University, Dalian, China; 2 Department of Nuclear Medicine, The First Affiliated Hospital of Dalian Medical University, Dalian, China

**Keywords:** Alzheimer’s disease, gut microbiota, positron emission tomography, insomnia disorder, brain pathology

## Abstract

**Objective:**

Previous studies on Alzheimer’s disease (AD) have focused on the relationships between brain pathology and gut microbiota, brain pathology and sleep, and sleep and gut microbiota, but no study has explored the relationship between these three factors. Therefore, we integrated these three factors into a unified framework and aimed to provide a reference for treating insomnia disorders (ID) in patients with AD.

**Patients/methods:**

The 65 patients diagnosed with AD were categorized into ID group (*n* = 30) and non-ID group (*n* = 35) according to the *Diagnostic and Statistical Manual of Mental Disorders, Fifth Edition*. Pittsburgh Sleep Quality Index (PSQI) was used to assess sleep quality. ^18^F-fluorodeoxyglucose (FDG)-positron emission tomography (PET) and ^18^F-florbetapir (AV45)-PET scan were performed. Fecal samples were analyses using 16S rRNA amplicon sequencing. Basic data, PET, and gut microbiota were compared between the ID and non-ID groups. Finally, the relationships among the data, with differences including PSQI, were analyses. All *p*-values were corrected using the False Discovery Rate (FDR) method to obtain *q*-values.

**Results:**

Data with significant differences (*p* < 0.05 or *q* < 0.05) included PSQI, left middle frontal cortex-FDG, left Broca’s area-FDG, right thalamus (rTh)-FDG, left thalamus (lTh)-FDG, *Roseburia*, *Prevotella 7*, and *Bifidobacterium*. However, no differences were found between groups in AV45-PET. In the ID group, PSQI scores were significantly correlated with rTh-FDG (*r* = −0.612, *q* < 0.05), lTh-FDG (*r* = −0.585, *q* < 0.05), and *Bifidobacterium* (*r* = −0.637, *q* < 0.05). Partial least squares structural equation modeling revealed that Thalamic-FDG exerted a partial mediating effect in the association between Bifidobacterium and PSQI scores.

**Conclusion:**

In AD patients with ID, there may be both a direct and an indirect association between *Bifidobacterium* and sleep quality, with thalamic glucose metabolism mediating the indirect association, indicating that treatments aimed at enhancing brain metabolism and probiotic supplementation may improve sleep quality in this population.

## 1 Introduction

Alzheimer’s disease (AD) is a leading cause of dementia among older adults, placing a significant burden on society and families, and has become a global health concern (1). Currently, the amyloid-β (Aβ) hypothesis remains one of the most widely accepted theories of AD pathogenesis. This hypothesis suggests that the abnormal accumulation and aggregation of Aβ peptides in the brain trigger a cascade of neurotoxic events, including the formation of tau tangles, synaptic dysfunction, and neuronal loss (2). The primary symptoms of AD include progressive cognitive decline, psychiatric and behavioral symptoms, and impairment in activities of daily living. Insomnia disorder (ID), one of the common psychiatric and behavioral symptoms in patients with AD, can lead to increased levels of Aβ in cerebrospinal fluid, which thereby exacerbates AD pathology. Additionally, ID disrupts the blood–brain barrier and impairs memory and synaptic function. Correcting ID could serve as a promising strategy for AD prevention and treatment (3).

Another widely recognized theory in AD is that the reciprocal influence between the brain pathological changes and gut microbiota mainly occurs through the gut-brain axis (GBA). This theory posits that there is a bidirectional interaction between the brain pathology and the gut microbiota (4). The brain can affect the gut microbiota through the autonomic nervous, immune, endocrine, and neurotransmitters. In turn, the gut microbiota can impact the brain through metabolites, immunity, neurotransmitters, and inflammation. A study has shown that injection of Aβ into the lateral ventricle of mice led to gut microbiota imbalance, an increase in pro-inflammatory factors, and inhibition of cholinergic anti-inflammatory pathways (5). In another animal trial, mice fed probiotics containing *Bifidobacterium* demonstrated increased ^18^F-fluorodeoxyglucose (FDG) uptake in brain tissue (6).

Furthermore, the GBA is involved in the relationship between ID and the gut microbiota. ID can contribute to gut microbiota disorders and digestive diseases, such as ulcers (7), Crohn’s disease (8), and irritable bowel syndrome (9). A study has shown that sleep deprivation can cause significant compositional and functional changes in gut microbiota (10). The imbalance of gut microbiota may, in turn, influence sleep quality through mechanisms such as the secretion of gamma-aminobutyric acid (GABA) and short-chain fatty acids (SCFAs) (11, 12) or by triggering systemic inflammation that further interferes with sleep (13). Supporting this link, Lee et al. found that participants consuming probiotics experienced improved sleep quality and reduced serum levels of interleukin-6, a key inflammatory marker (14). Given the intimate connection between sleep and AD, investigating the role of the GBA in AD patients with ID is of great significance.

Long-term reliance on hypnotics such as benzodiazepines to treat ID in patients with AD not only causes cognitive impairment but also fails to fundamentally solve the problem (15). Therefore, it is necessary to search for new therapeutic targets. However, previous studies on AD have focused on the relationships between brain pathology and gut microbiota, brain pathology and sleep, and sleep and gut microbiota, but no study has explored the relationship between these three factors. Therefore, we integrated these three factors into a unified framework and aimed to provide a reference for treating ID in patients with AD.

Our study is the first to apply partial least squares structural equation modeling (PLS-SEM) to explore the mediating effect within the GBA in AD patients with ID. Through these multi-dimensional and comprehensive research approaches, we expect to reveal underlying associations unaddressed in previous studies, provide new targets and strategies for treating ID in this population, and thus further fill the gaps in existing research.

## 2 Methods

### 2.1 Participants

Inclusion criteria were as follows: patients admitted to the First Affiliated Hospital of Dalian Medical University between 2019 and 2023 who were diagnosed with probable AD according to the National Institute of Neurological and Communicative Disorders and Stroke and Alzheimer’s Disease and Related Disorder Association Alzheimer’s Criteria revised in 2011 (16) and for whom it was confirmed through medical records, patient self-reports, or other reliable means that the onset of cognitive impairment preceded the onset of sleep disorder. Within 1 month, the Mini-Mental State Examination (MMSE) (used to evaluate cognitive function) (17), the Hamilton Depression Rating Scale (HAMD) and Hamilton Anxiety Rating Scale (HAM-A) (used to measure depressive and anxious symptoms, respectively), and the Pittsburgh Sleep Quality Index (PSQI) (18) (used to evaluate sleep quality) were completed; stool samples were collected; and FDG-positron emission tomography (PET) and ^18^F-florbetapir (AV45)-PET were performed. The participants were divided into ID and non-ID groups. The ID group was diagnosed based on the Diagnostic and Statistical Manual of Mental Disorders, Fifth Edition (DSM-5) criteria.

Exclusion criteria were as follows: (1) family history of AD; (2) other types of dementia or other diseases affecting cognitive function (e.g., severe cerebrovascular disease, brain tumors, Parkinson’s disease, multiple sclerosis, epilepsy, encephalitis, human immunodeficiency virus infection, and *Treponema pallidum* infection); (3) alcoholism or use of drugs affecting cognitive function; (4) mental disorders (e.g., schizophrenia, autism, moderate/severe anxiety disorder, and moderate/severe depression); (5) digestive diseases (e.g., gastrointestinal inflammation, irritable bowel syndrome, and diarrhea within 1 month before sample collection); (6) daytime sleepiness or other symptoms or diseases affecting sleep (e.g., tumors, chronic pain, cardiac insufficiency, chronic obstructive pulmonary disease, hepatic and renal insufficiency, thyroid dysfunction, and infectious diseases); (7) use of zopiclone within 2 weeks, and use of, antibiotics, probiotics, hormones, immunosuppressant’s, or similar medications within 4 weeks before sample collection, PET/computed tomography (CT), or questionnaires; and (8) special dietary habits (including vegetarianism, ketogenic diet, and intermittent fasting).

### 2.2 Sleep quality assessment

The PSQI was used to assess the sleep quality of the participants, including 19 items and seven sleep quality dimensions (sleep quality, sleep time, sleep duration, sleep efficiency, sleep disorder, hypnotic drugs, and daytime dysfunction). Each item was scored on a specific scale, with the total score ranging from 0 to 21.

### 2.3 PET image acquisition

All participants underwent PET/CT scans (Biograph 64 PET/CT; Siemens Shanghai Medical Equipment Ltd., Shanghai, China), including ^18^F-FDG-PET and ^18^F-AV45-PET to assess cerebral glucose metabolism and Aβ deposition, respectively. During the scans, a CT scan was first performed with a tube voltage of 120 kV, a current of 300 mAs, a pitch of 0.8, a reconstruction slice thickness of 3 mm, and a reconstruction matrix of 512 × 512. Subsequently, a bed-based PET acquisition was conducted for 10 min, with CT images used for attenuation correction (slice thickness 3 mm, consistent with that of the CT images). For PET reconstruction, the True X algorithm was employed, with 3 iterations, 21 subsets, Gaussian filtering (FWHM = 4 mm), and a matrix size of 336 × 336. For the FDG-PET, participants were required to follow a bland diet 1 day before the examination, fast for 4–6 h before the scan, and hold all medications on the day of the examination, with fasting blood glucose levels required to be <140 mg/dL. They rested in a quiet, dark environment for 60 min before the intravenous injection of the radiopharmaceutical at a dose of 0.15 mCi/kg. The AV45-PET required no special preparation except for the administration of 10 mCi of the radiotracer. A minimum interval of 3 days was maintained between the two PET/CT procedures. PET/CT images were processed using Neuro Q software, which automatically analyses 47 brain regions to derive standardized uptake value ratios (SUVRs) and pixel values. For each brain region, the pixel value was normalized by subtracting the mean SUVR of the database template and dividing by the template’s standard deviation to calculate the *Z*-score for radiopharmaceutical uptake. FDG-PET was considered positive when its *Z*-score was ≤1.65, indicating low glucose metabolism. AV45-PET was considered positive when its *Z*-score was ≥1.65, indicating the presence of Aβ deposition. The AV45-PET images were evaluated by two experienced nuclear medicine physicians (blinded to clinical data) using a binary visual assessment method to determine the presence of Aβ deposition.

### 2.4 Gut microbiota analyses

The patients’ fecal samples were collected using a disposable medical fecal tube and frozen in liquid nitrogen within 1 h after collection, and then transported to a refrigerator at −80 °C for storage. DNA was extracted from the feces. Specifically, 1,000 μL CTAB lysis buffer and 20 μL lysozyme were added to a 2.0 mL EP tube, then an appropriate amount of the fecal sample was added to the lysis buffer, followed by a water bath at 65 °C for 2 h (with inversion and mixing several times during the period to ensure sufficient lysis of the sample). After centrifugation at 12,000 rpm for 10 min, 950 μL of the supernatant was collected and mixed with an equal volume of phenol (pH 8.0):chloroform:isoamyl alcohol (25:24:1) by inversion, then centrifuged at 12,000 rpm for 10 min. The supernatant was taken and mixed with an equal volume of chloroform: isoamyl alcohol (24:1) by inversion, followed by centrifugation at 12,000 rpm for 10 min. The resulting supernatant was transferred to a 1.5 mL centrifuge tube, and isopropanol (with a volume of 3/4 of the supernatant) was added; the mixture was shaken up and down and placed at −20 °C for precipitation. After centrifugation at 12,000 rpm for 10 min, the liquid was poured out (taking care not to pour out the precipitate), and the precipitate was washed twice with 1 mL of 75% ethanol; the remaining small amount of liquid could be collected by centrifugation again and then aspirated with a pipette tip. The precipitate was dried in a ultra-clean workbench or at room temperature (avoiding excessive drying of the DNA sample as it would be difficult to dissolve subsequently), then 51 μL ddH2O was added to dissolve the DNA sample (incubation at 55–60 °C for 10 min could be performed if necessary to assist dissolution), and finally 1 μL RNase A was added to digest RNA, followed by placement at 37 °C for 15 min. Then the 16S RNA gene was amplified through polymerase chain reaction (PCR). The amplified region was 16SV34, and the primer sequences were CCTAYGGGRBGCASCAG and GGACTACNNGGGTATCTAAT. Subsequently, 15 μL Phusion High-Fidelity PCR Master Mix (New England Biolabs), 0.2 μM primer, and 10 ng of genomic DNA template were added to all PCR mixtures. Initial denaturation was performed at 98 °C (1 min), followed by 30 cycles at 98 °C (10 s), 50 °C (30 s), and 72 °C (30 s), and finally maintained at 72 °C (5 min). Qualitative PCR products were purified using magnetic beads and quantified through enzyme labeling. For 16S sequencing library preparation targeting the 16SV34 region (i.e., V3–V4 region), the specific protocol was as follows: the PCR system for library construction used 10–30 ng of template DNA, primers including 338F (5′-ACTCCTACGGGAGGCAGCAG-3′) and 806R (5′-GGACTACHVGGGTWTCTAAT-3′), and high-fidelity enzymes (FastPfu); the PCR conditions were set as follows: initial denaturation at 95 °C for 3 min, followed by 25–28 cycles of denaturation at 95 °C for 30 s, annealing at 55 °C for 30 s, and extension at 72 °C for 30 s, and finally a final extension step at 72 °C for 5 min. After the library was constructed, PE250 sequencing was performed using NovaSeq6000. The sample data were separated from the raw sequencing data based on the barcode and PCR-amplified primer sequences. After truncating the barcode and primer sequences, reads from each sample were spliced using FLASH (version 1.2.11). Chimeric sequences were removed, and the effective tags were denied using the DADA2 module in the QIIME2 (Version QIIME2-202202) software to obtain the final amplicon sequence variants and a characterization table. The QIIME2 software was used for species annotation.

### 2.5 Statistical analyses

Basic clinical data, FDG-PET, and AV45-PET of each brain region were compared between the ID and non-ID groups. For categorical variables, the chi-square test or Fisher’s exact test was applied. Normally distributed measurement data were analyses using the independent samples *t*-test, while non-normally distributed measurement data were analyses using the Mann–Whitney *U* test. QIIME software was used to calculate gut microbiota diversity (Chao1, Shannon, and Simpson) and β diversity (Bray–Curtis distance). Linear discriminant analysis (LDA) effect size (LEfSe) was used to identify microbial taxa that significantly differed between the ID and non-ID groups, with a threshold LDA score of >3.5 (19). The gut microbiota with significant differences were analyses using the Mann–Whitney *U* test or the independent samples *t*-test. The relationships between PET, gut microbiota, and PSQI scores were analyses using Pearson’s or Spearman’s correlation tests, depending on data distribution. Based on these correlation results, PLS-SEM was used to analyses the mediating effect in the gut-brain axis. Additionally, binary logistic regression was used to separately analyses, the predictive efficacy of gut microbiota, PET, and their combination on the occurrence of ID, and then the advantages and disadvantages of the three models were compared. Finally, we established a LASSO regression model to further explore whether PSQI and/or gut microbiota can serve as predictors of PET. All *p*-values were corrected using the False Discovery Rate (FDR) method to obtain q-values. Statistical significance was set at *p*-value <0.05 (both sides) or FDR *q*-value <0.05 (both sides). IBM SPSS 25.0, Smart PLS 4.1, Python 3.10.9, and QIIME 2 were used for analysis.

## 3 Results

### 3.1 Study population

**Figure 1:**
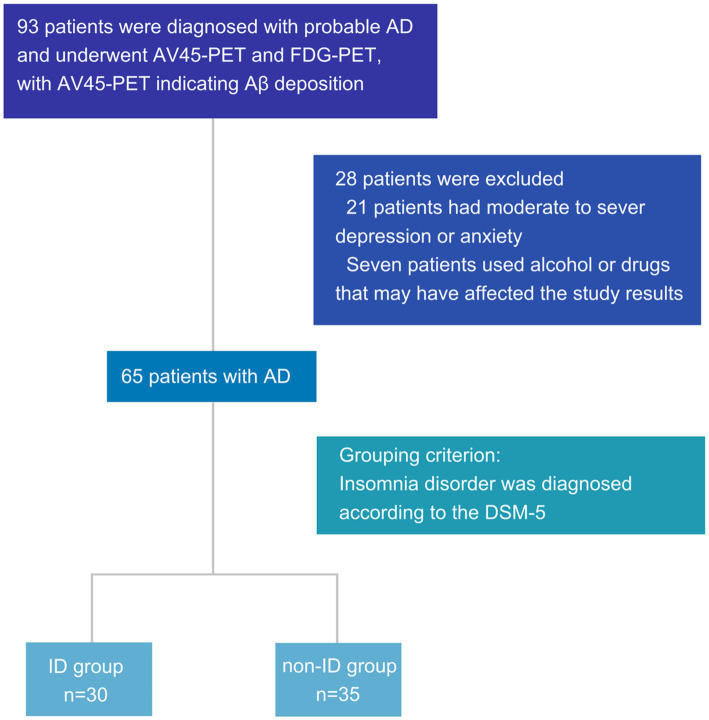
Flowchart of patient inclusion. AD, Alzheimer’s disease; AV45-PET, ^18^F-florbetapir positron emission tomography; FDG-PET, ^18^F-fluorodeoxyglucose positron emission tomography; PSQI, Pittsburgh sleep quality index; DSM-5, diagnostic and statistical manual of mental disorders, 5th edition; ID, insomnia disorder; Aβ, amyloid-β.

Overall, 93 patients were diagnosed with probable AD and underwent AV45-PET and FDG-PET, with AV45-PET indicating Aβ deposition. Among the patients, 21 were excluded for moderate to severe depression or anxiety, and seven were excluded for using alcohol or drugs that may have affected the study results. Finally, 30 and 35 patients were screened in the ID and non-ID groups, respectively, according to the grouping criteria (Figure 1).

The average age of the ID group was 70.57 ± 6.73 years, including 11 males (36.7%), while the non-ID group displayed an average age of 70.37 ± 6.84 years, with 16 males (45.7%). There were no differences in terms of sex, age, disease duration, years of education, body mass index (BMI), left handedness, MMSE, HAMD, HAM-A, and medical history between the ID and non-ID groups, which proved the comparability of the two groups at baseline; however, there were significant differences in PSQI, which is used to evaluate sleep quality, indicating that the grouping was reasonable (Table 1).

**Table 1:** Comparison of patient demographics, clinical features, and neuropsychological scales between the ID and non-ID groups.

Characteristics	ID group (*n* = 30)	Non-ID group (*n* = 35)	*p*-value	Effect size
Male (%)	11 (36.7)	16 (45.7)	0.461	−0.090
Age, years	70.57 ± 6.73	70.37 ± 6.84	0.908	0.029
Duration, years	6.30 ± 2.81	5.46 ± 3.18	0.265	0.280
Education, years	9.13 ± 2.24	9.37 ± 2.41	0.712	−0.102
BMI	37.70 ± 5.18	39.65 ± 5.41	0.223	−0.367
Diabetes (%)	5 (16.7)	6 (17.1)	0.959	−0.005
Hypertension (%)	10 (33.3)	12 (34.3)	0.936	−0.010
Smoking (%)	6 (20.0)	6 (17.1)	0.767	0.029
Left-handedness (%)	2 (6.7)	4 (11.4)	0.817	−0.048
MMSE	12.97 ± 3.76	13.34 ± 2.86	0.984	−0.114
HAM-A	7.93 ± 2.32	8.20 ± 2.08	0.659	−0.122
HAMD	9.80 ± 2.73	10.14 ± 2.03	0.685	−0.144
PSQI	12.80 ± 2.09	3.34 ± 1.28	<0.001^**^	5.554

For categorical variables, effect size was quantified using the risk difference. For continuous variables, Cohen’s d was used to measure effect size. ID, insomnia disorder; BMI, body mass index; MMSE, mini-mental state examination; HAM-A, Hamilton anxiety rating scale; HAMD, Hamilton depression rating scale; PSQI, Pittsburgh sleep quality index. ^**^p < 0.01 indicates statistical significance.

### 3.2 Intergroup differences in cerebral PET imaging features

**Figure 2:**
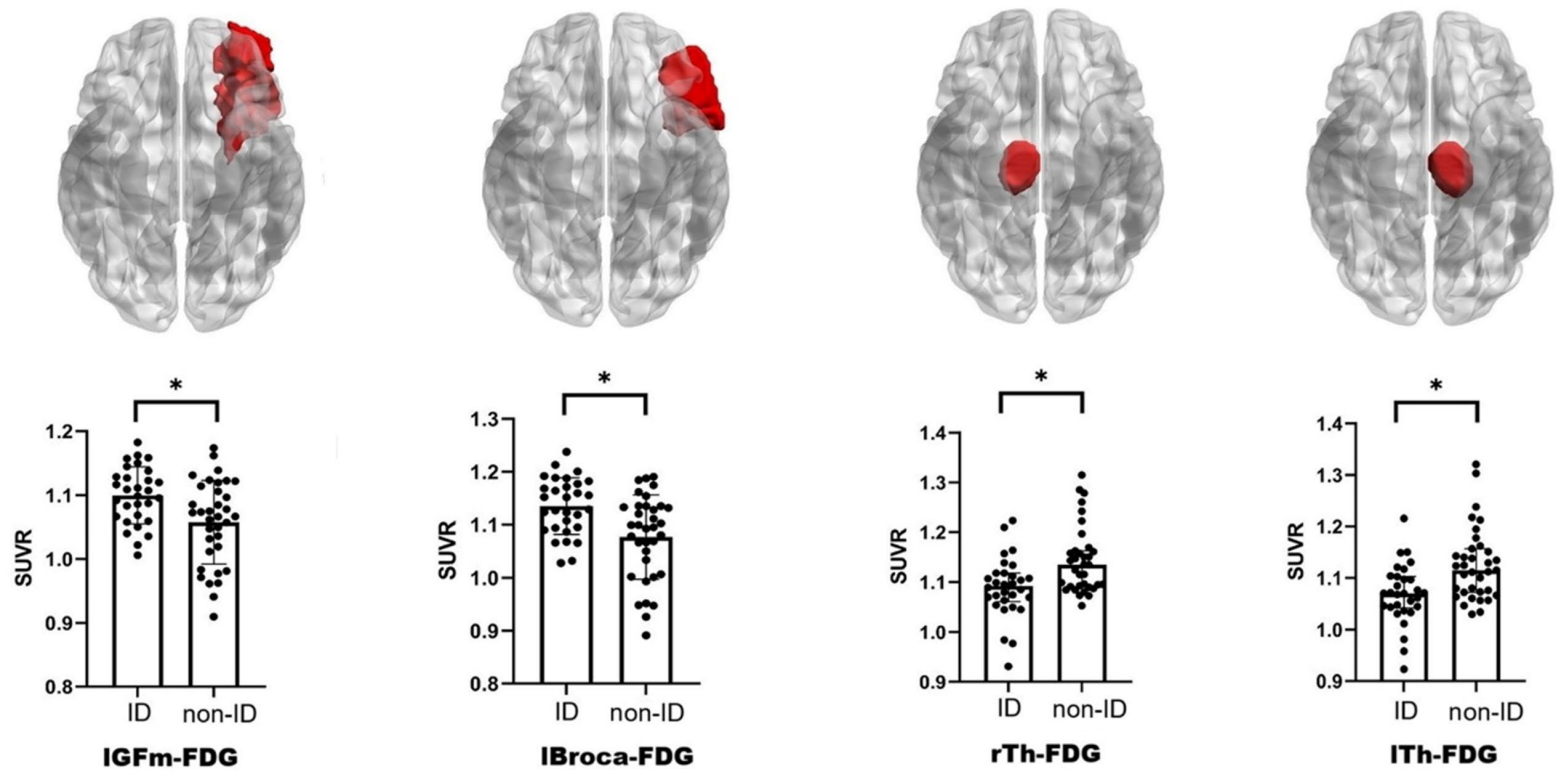
Schematic diagram and scatter bar chart of brain regions with differences. The brain diagram follows radiological convention, and the brain regions colored red indicate those exhibiting significant differences in FDG-PET. ID, insomnia disorder; FDG-PET, ^18^F-fluorodeoxyglucose positron emission tomography; lGFm, left middle frontal cortex; lBroca, left Broca’s area; lTh, left thalamus; rTh, right thalamus; SUVR, standardized uptake value ratio. ^*^q < 0.05 indicates statistical significance.

FDG-PET in the left middle frontal cortex (lGFm) (*t* = 3.045, *q* = 0.035), left Broca’s area (lBroca) (*t* = 3.402, *q* = 0.024), left thalamus (lTh) (*q* = 0.024), and right thalamus (rTh) (*q* = 0.031) differed between the ID and non-ID groups (*q* < 0.05), but no differences were observed in other brain regions (Figure 2; Supplementary Table 1). No differences were found in AV45-PET between the ID and non-ID groups (Supplementary Table 2).

### 3.3 Intergroup differences in gut microbiota

#### 3.3.1 Diversity analysis of gut microbiota

No significant differences were observed in α diversity (Chao1, Shannon, and Simpson indices), which measures species richness and evenness, or in β diversity (Bray–Curtis distance), which evaluates compositional differences between samples, of gut microbiota between the ID and non-ID groups (Table 2).

**Table 2:** Comparison of gut microbiota diversity between the ID and non-ID groups.

Diversity indices	ID group (*n* = 30)	Non-ID group (*n* = 35)	*p*-value
Chao1	232.98 ± 73.25	246.09 ± 75.52	0.482
Shannon	5.16 ± 0.82	5.33 ± 0.63	0.502
Simpson	0.92 ± 0.07	0.93 ± 0.04	0.258
Bray–Curtis	0.83 ± 0.14	0.82 ± 0.14	0.443

ID, insomnia disorder. All *p*-values are greater than 0.05, indicating no statistically significant differences.

#### 3.3.2 Analysis of taxonomic differences

**Figure 3:**
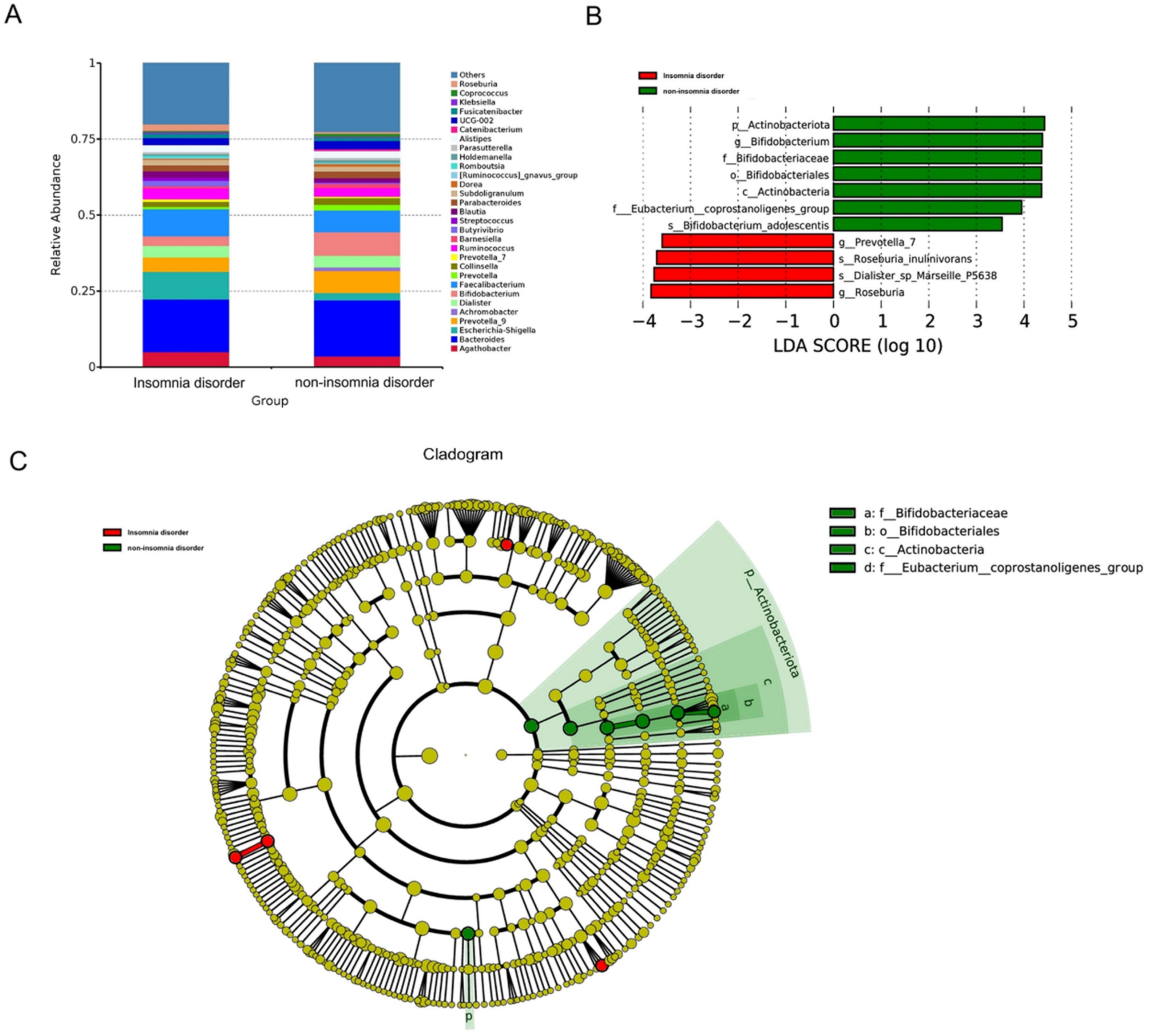
The differential presentation of gut microbiota. **(A)** The top 30 relative abundance of microbiota at the genus level in the ID and non-ID groups. **(B)** The histogram displays the gut microbiota that had significant differences in abundance at various taxonomic levels, namely phylum, class, order, family, and genus, between the ID group and the non-ID group. Red indicates enrichment in the ID group, and green indicates enrichment in the non-ID group. These differences were identified via LEfSe analysis and Mann–Whitney *U* test (LDA score > 3.5, *p* < 0.01). **(C)** In the evolutionary branch diagram, from the innermost to the outermost layers correspond to kingdom, phylum, class, order, family, genus, and species. Yellow indicates no statistical difference, while red indicates enrichment in the ID group, and green indicates enrichment in the non-ID group.

However, the LEfSe analysis and the Mann–Whitney *U* test identified significantly higher abundances in the non-ID group at multiple taxonomic levels, including *Bifidobacterium adolescentis* (species) (LDA = 3.540, *p* = 0.016), *Bifidobacterium* (genus) (LDA = 4.329, *p* = 0.006), *Bifidobacterium*ceae (family) (LDA = 4.322, *p* = 0.006), *Eubacterium coprostanoligenes group* (family) (LDA = 3.951, *p* = 0.008), *Bifidobacterium*les (order) (LDA = 4.322, *p* = 0.006), *Actinobacteria* (class) (LDA = 4.331, *p* = 0.004), and *Actinobacteriota* (phylum) (LDA = 4.398, *p* = 0.004). The gut microbiota enriched in the ID group included *Dialister* sp. *Marseille P5638* (species) (LDA = 3.540, *p* = 0.016), *Roseburia inulinivorans* (species) (LDA = 3.711, *p* = 0.011), *Prevotella 7* (genus) (LDA = 3.596, *p* = 0.0498), *Roseburia* (genus) (LDA = 3.829, *p* = 0.008) (Figure 3).

### 3.4 PET-microbiota-PSQI relationships

**Figure 4:**
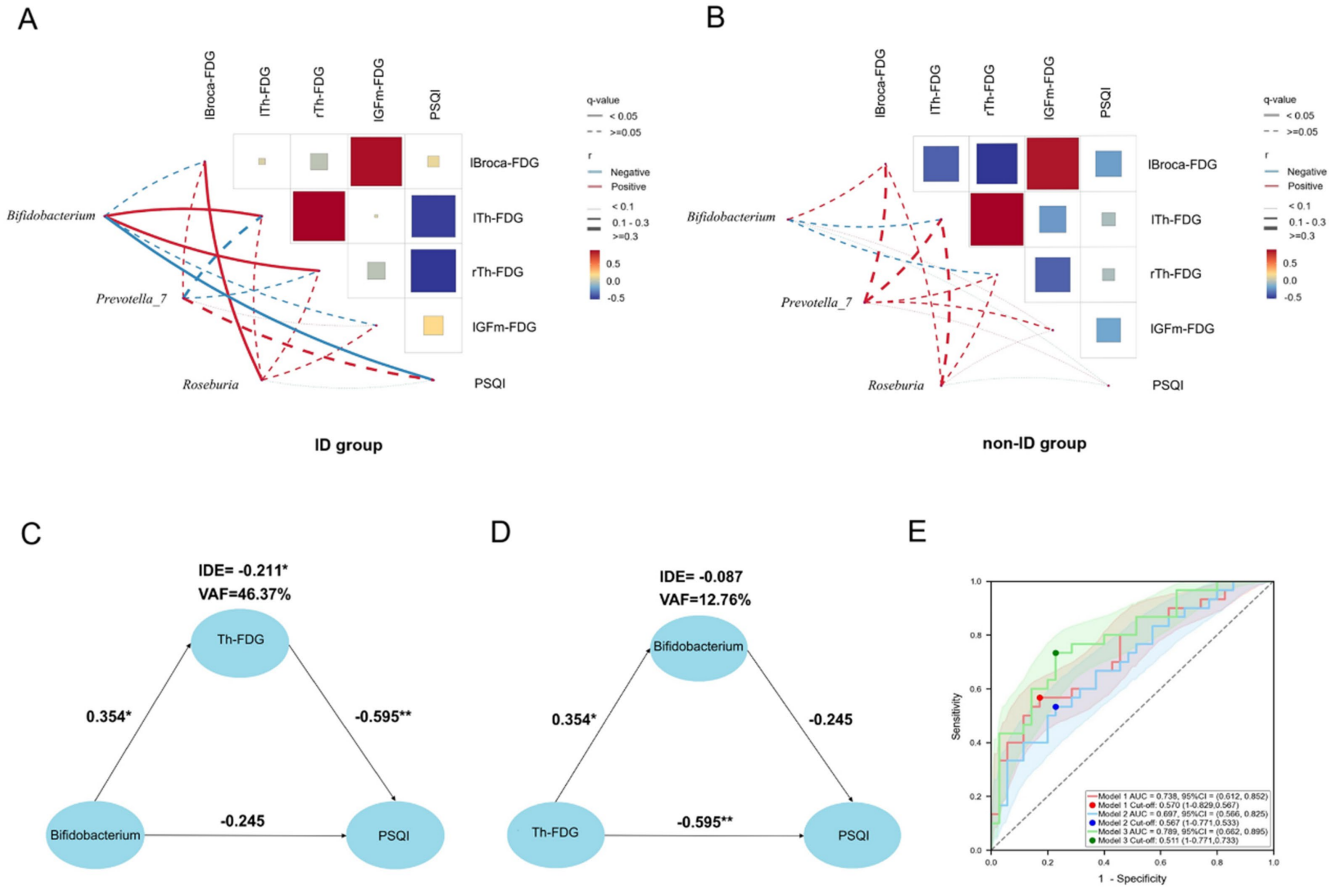
PET-microbiota-PSQI relationships. **(A,B)** Correlation matrix heat maps of gut microbiota with differences (*Bifidobacterium*, Prevotella_7, *Roseburia*), FDG uptake of brain regions with differences (lBroca, lTh, rTh, lGFm), and PSQI scores in the ID and non-ID groups. The size and color of the squares, and the thickness of the lines, represent the correlation coefficient (*r*) values. The red line represents a positive correlation, the blue line represents a negative correlation, the solid line represents *q* < 0.05, and the dotted line represents *q* ≥ 0.05. **(C)** PLS-SEM structural model with Th-FDG as the mediating variable. PLS-SEM found that in AD patients with ID, *Bifidobacterium* affects PSQI by regulating the level of Th-FDG. Each path is marked with a path coefficient. **(D)** PLS-SEM structural model with *Bifidobacterium* as the mediating variable. The impact of Th-FDG on the PSQI does not occur through the mediating effect of *Bifidobacterium*, but has a direct effect on the PSQI. **(E)** The area under curve of the three models. All models adopted the binary logistic model, with the outcome variable being whether the patients had ID. The red line represents model 1 (independent variable is Th-FDG), the blue line represents model 2 (independent variable is relative abundance of *Bifidobacterium*), and the green line represents model 3 (independent variables are Th-FDG and relative abundance of *Bifidobacterium*). The shaded areas corresponding to the respective colors are the confidence intervals of the models. ^*^p < 0.05 indicates that the path coefficient is statistically significant. ^**^p < 0.01 indicates that the path coefficient is highly statistically significant. PSQI, Pittsburgh Sleep Quality Index; Th, thalamus; IDE, Indirect Effect; VAF, Variance Accounted For; FDG, ^18^F-fluorodeoxyglucose positron emission tomography; lGFm, left middle frontal cortex; lBroca, left Broca’s area; lTh, left thalamus; rTh, right thalamus; ID, insomnia disorder; r, correlation coefficient.

To further explore the associations among variables with significant group differences, we conducted a multiple correlation analysis of PSQI scores, FDG-PET, and gut microbiota at the genus level in the ID and non-ID groups, respectively. In the ID group, *Bifidobacterium* was positively correlated with rTh-FDG (*r* = 0.509, *q* = 0.009) and lTh-FDG (*r* = 0.519, *q* = 0.008), and was negatively correlated with PSQI score (*r* = −0.637, *q* < 0.001). lBroca-FDG was positively correlated with lGFm-FDG (*r* = 0.796, *q* < 0.001). PSQI scores were negatively correlated with rTh-FDG (*r* = −0.612, *q* < 0.001) and lTh-FDG (*r* = −0.585, *q* = 0.005) (Figure 4A). In the non-ID group, lBroca-FDG was positively correlated with lGFm-FDG (*r* = 0.859, *q* < 0.001), and was negatively correlated with rTh-FDG (*r* = −0.528, *q* = 0.005). rTh-FDG was positively correlated with lTh-FDG (*r* = 0.897, *q* < 0.001) (Figure 4B). The data with multiple correlations were rTh-FDG, lTh-FDG, PSQI scores, and *Bifidobacterium* in the ID group. The correlation coefficients for both groups are presented in Supplementary Tables 3, 4.

### 3.5 The mediating effect of thalamic glucose metabolism

The primary objective of this study was to explore the ID in patients with AD and uncover potential therapeutic targets. Meanwhile, to clarify the specific pathway of GBA through which gut microbiota affects the sleep quality of AD patients with ID, we regarded the symptoms as the dependent variable and the gut microbiota, which is at the other end of GBA, as the independent variable. The mediating variable is thalamic (Th)-FDG, including right thalamic (rTh)-FDG and left thalamic (lTh)-FDG. We used PLS-SEM to analyses whether *Bifidobacterium* affected PSQI through the mediating effect of thalamic glucose metabolism. The results showed that there was no direct effect of *Bifidobacterium* on PSQI (Path coefficient, *β* = −0.245, *p* = 0.07), while Th-FDG had a complete mediating effect in the influence of *Bifidobacterium* on PSQI (Indirect Effect, IDE = −0.211; Variance Accounted For, VAF = 46.37%, *p* < 0.05). This indicates that *Bifidobacterium* impacted the sleep quality of AD patients with ID by interfering with the thalamic glucose metabolism level. The mediating effect of the thalamic glucose metabolism level can explain 46.37% of the changes in PSQI (Figure 4C; Supplementary Tables 5, 6). To verify whether *Bifidobacterium* has a mediating effect in the influence of Th-FDG on PSQI, we took PSQI as the dependent variable, Th-FDG as the independent variable, and *Bifidobacterium* as the mediating variable. The results showed that Th-FDG had a direct effect on PSQI (*β* = −0.595, *p* < 0.01), while *Bifidobacterium* had no mediating effect (IDE = −0.087, *p* = 0.135) (Figure 4D; Supplementary Tables 7, 8).

### 3.6 Comparison of logistic regression models with ID as the outcome variable

Previous research predominantly focused on exploring single relationships. In contrast, our study adopted a multi-dimensional and comprehensive approach to determine whether a comprehensive investigation of the combined effects of gut microbiota and brain pathological changes on ID in patients with AD offered distinct advantages over examining the effects of either in isolation. To achieve this, we established three models with ID as the binary outcome variable and analyses them. Considering collinearity between rTh-FDG and lTh-FDG (*r* = 0.818), we calculated their average value to represent thalamic glucose metabolism, obtaining Th(avg)-FDG (Figure 4E).

In Model 1, the independent variable was Th(avg)-FDG; in Model 2, it was the relative abundance of *Bifidobacterium*; and in Model 3, both were included. In terms of accuracy, Model 1 achieved 0.631, Model 2 0.646, and Model 3 0.738 (with Model 3 exhibiting the highest). For AUC, Model 1 scored 0.738, Model 2 0.697, and Model 3 0.789 (Model 3 had the highest diagnostic efficiency). McFadden’s R-squared values were 0.140 (Model 1), 0.082 (Model 2), and 0.198 (Model 3). AIC and BIC values were 81.19/85.53 (Model 1), 86.33/90.68 (Model 2), and 77.95/84.47 (Model 3). Lower AIC and BIC values indicate a model balances goodness-of-fit and complexity better, and Model 3 had the lowest values, reflecting superior performance. Further, model fit was assessed using the Hosmer–Lemeshow (H-L) goodness-of-fit test: the univariate Model 1 yielded an H-L test-derived chi-square value of 5.658 (*p* = 0.685); the univariate Model 2 an H-L test-derived chi-square value of 3.162 (*p* = 0.924); and the multivariate Model 3 an H-L test-derived chi-square value of 5.864 (*p* = 0.662). The high *p*-values indicate no significant differences between observed and predicted values across models, confirming good fit. Additionally, collinearity in Model 3 was evaluated using the Variance Inflation Factor (VIF): both variables [Th(avg)-FDG and *Bifidobacterium*] had a VIF of 1.018, far below the threshold of 5, indicating no severe collinearity between Th(avg)-FDG and *Bifidobacterium*. It was concluded that Model 3 had the optimal performance, strongly implying that a comprehensive study outperformed that on single relationships.

### 3.7 Predictive analysis of FDG uptake using PSQI and Bifidobacterium as predictors

We undertook a preliminary exploration of whether PSQI and/or *Bifidobacterium* could serve as predictors of Th-FDG. The LASSO regression model identified significant predictors associated with rTh-FDG and lTh-FDG, focusing on minimizing prediction error through variable selection in the ID group.

**Figure 5:**
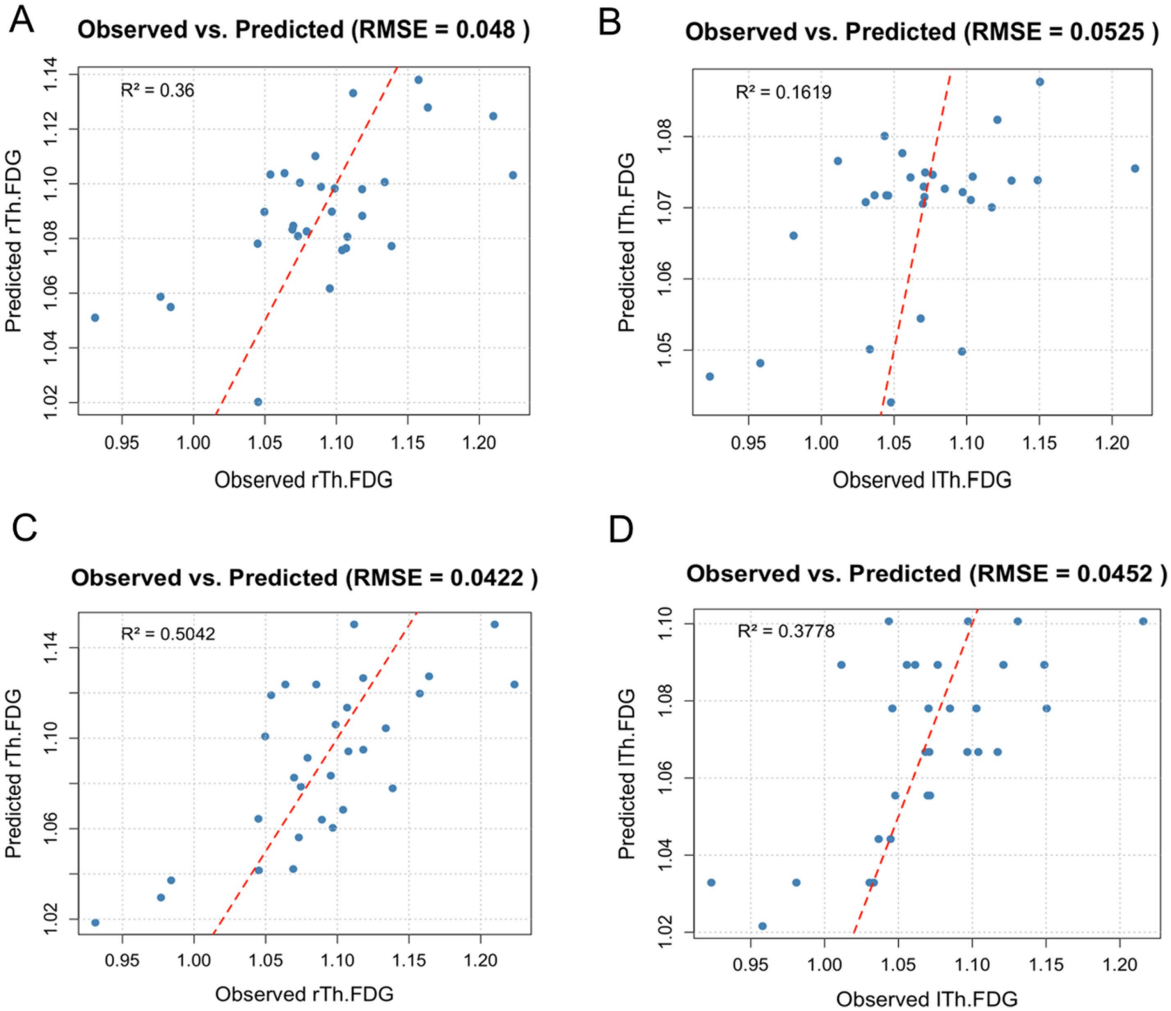
Observed versus predicted FDG uptake in right and left thalamus regions. This scatter plot illustrates the observed versus predicted FDG uptake in the right and left thalamus regions (rTh-FDG and lTh-FDG) based on the lasso regression models, which included demographic and lifestyle factors, as well as either *Bifidobacterium* or PSQI. Each point represents an individual data entry, with a red diagonal reference line indicating a perfect prediction fit. Model performance is reflected by root mean squared error (RMSE) and *R*^2^ values. **(A,B)** Observed versus predicted FDG uptake in right and left thalamus regions when *Bifidobacterium* was included in the model. **(C,D)** Observed versus predicted FDG uptake in right and left thalamus regions when PSQI was included in the model.

When *Bifidobacterium* was included in the model, the rTh-FDG model retained sex, smoking, disease duration, and *Bifidobacterium* as significant predictors, with coefficients of −0.0009, −0.031, −0.006, and 0.132, respectively. Other variables, such as age, BMI, hypertension, diabetes, and handedness, were excluded, suggesting minimal impact on rTh-FDG prediction (Figure 5A). Similarly, for the lTh-FDG outcome, the model retained smoking, disease duration, and *Bifidobacterium* as significant predictors, with coefficients of −0.0009, −0.023, and 0.071, respectively (Figure 5B).

When PSQI was included, the rTh-FDG model retained sex, smoking, disease duration, and PSQI as significant predictors, with coefficients of 0.004, −0.005, −0.004, and −0.013, respectively (Figure 5C). For the lTh-FDG model, smoking, disease duration, and PSQI were also significant, with coefficients of −0.0009, −0.023, and 0.071, respectively (Figure 5D). Notably, the lTh-FDG outcome model retained only PSQI as a significant predictor, with a coefficient of −0.011.

The model demonstrated a high predictive *R^2^*, explaining approximately 16%–50% of the variance in FDG uptake. Additionally, it achieved a low Root Mean Squared Error (RMSE), reflecting minimal average prediction error and overall fit.

### 3.8 Sample size validation and sensitivity analysis

We calculated the required sample size using G*Power 3.1 software, based on data from the PSQI and thalamic glucose metabolism, with α set at 0.05 and β at 0.2. Among the calculations, the rTh-FDG corresponded to the largest minimum required sample size (*n* = 48), meaning each group needed to include at least 24 participants. Given that we ultimately enrolled 30 patients in the ID group and 35 in the non-ID group—both exceeding the 24-patient minimum per group—this confirms that the sample size of our study is sufficient to ensure adequate statistical power for detecting potential between-group differences in the key indicators (e.g., PSQI, FDG-PET).

To verify the robustness of intergroup differences in *Bifidobacterium*, and given that baseline data showed no differences between the two groups, we constructed a simplified linear model. This model was used to validate the effect of grouping on *Bifidobacterium* via an alternative approach, while enhancing the interpretability of the results. To ensure *Bifidobacterium* data met the model assumptions and better reflected its data characteristics, we performed a Centered Log-Ratio (CLR) transformation on the data. The CLR-transformed *Bifidobacterium* was set as the dependent variable, and grouping as the independent variable. The resulting model equation was: *Bifidobacterium* = 3.8037–1.1642 × Grouping, with a *p*-value of 0.023 for the “Grouping” variable. This indicates that the “Grouping” variable exerts a significant effect on CLR-transformed *Bifidobacterium* levels. Previously, we had compared the distribution of *Bifidobacterium* between the two groups using the Mann–Whitney *U* test. The linear model validated the effect of grouping on *Bifidobacterium* from an alternative perspective, and the two sets of results mutually corroborated each other.

We performed CLR transformation on gut microbiota, and then validated the subset of previously identified multiple correlations. The results showed that in the ID group, *Bifidobacterium* was positively correlated with rTh-FDG (*r* = 0.516, *q* = 0.013) and lTh-FDG (*r* = 0.596, *q* = 0.003), and negatively correlated with PSQI score (*r* = −0.614, *q* = 0.002). Additionally, PSQI scores were negatively correlated with rTh-FDG (*r* = −0.612, *q* = 0.002) and lTh-FDG (*r* = −0.585, *q* = 0.003) (Table 3). The findings indicate that the results of multiple correlation analyses performed after CLR transformation are highly consistent with those from analyses without transformation, in terms of the direction of correlations, the magnitude of correlation coefficients, and other relevant aspects.

**Table 3:** Multiple correlation analysis of the ID group following centered log-ratio (CLR) transformation.

Variables		*r*	*p*-value	*q*-value
*Bifidobacterium*	lTh-FDG	0.596	<0.001	0.003^**^
	rTh-FDG	0.516	0.004	0.013^*^
	PSQI	−0.614	<0.001	0.002^**^
lTh-FDG	rTh-FDG	0.818	<0.001	<0.001^**^
	PSQI	−0.585	<0.001	0.003^**^
rTh-FDG	PSQI	−0.612	<0.001	0.002^**^

FDG, ^18^F-fluorodeoxyglucose positron emission tomography; lTh, left thalamus; rTh, right thalamus; *r*, correlation coefficient. ^*^q < 0.05 indicates a significant correlation. ^**^q < 0.01 indicates a highly significant correlation.

We performed a mediation effect analysis on the CLR-transformed relative abundance of *Bifidobacterium*. The results showed that Th-FDG exerted a partial mediating effect in the relationship between *Bifidobacterium* and the PSQI, with an IDE of −0.227, a VAF of 34.76%, and statistical significance (*p* = 0.008). Additionally, *Bifidobacterium* had a direct effect on PSQI (Path coefficient, *β* = −0.426, *p* = 0.001).

## 4 Discussion

Our study found no difference in AV45-PET between the ID and non-ID groups, suggesting that Aβ deposition does not affect sleep in patients with AD. Likewise, a large sample study conducted by Benedict et al. on a local population found that there was no difference in Aβ levels between patients with and without sleep disturbance, and even after several years of follow-up, no difference was found (20). We believe that the symptoms of patients with AD are not only affected by the level of Aβ deposition, but also by the structure of Aβ. The heterogeneity of Aβ leads to the heterogeneity of symptoms of AD (21), that is, patients with similar Aβ deposition levels may exhibit different symptoms. Specifically, Aβ can be classified into monomers, oligomers, and fibrillary aggregates. In patients with AD, excessive Aβ monomers can transform into cytotoxic oligomers and fibrillary aggregates. Among them, Aβ oligomers play a central role in the pathology of AD. They can induce neurotoxicity and synaptic loss, while Aβ fibrillary aggregates mainly form senile plaques. AV45-PET primarily detects fibrillary Aβ plaques, and the negative result obtained in this study indicates that fibrillary Aβ burden is not correlated with insomnia in this AD cohort. Considering the limitations of AV45-PET in detecting soluble Aβ oligomers—which play a key role in synaptic toxicity—future studies may explore the relationship between Aβ with different structural forms and sleep (22).

The relative abundance of *Bifidobacterium* was negatively correlated with PSQI scores in the ID group. This suggested that higher *Bifidobacterium* abundance corresponded to lower PSQI scores and better sleep quality. Animal experiments conducted by Bowers et al. (23) showed that *Bifidobacterium* in sleep-disturbed mice was reduced. They attributed this reduction to disturbed sleep, which affects the hypothalamic–pituitary–adrenal axis in mice, increases inflammation, and alters the gut microbiota (24) used data from the MiBio Gen consortium and the UK Biobank to determine that *Bifidobacterium* exhibited a significant causal relationship with sleep. They considered that the butyrate produced by *Bifidobacterium* reduced inflammation, which interacted with sleep. In the study of Lee et al. (14), participants’ sleep quality improved after taking probiotics containing *Bifidobacterium*, and a decrease in serum levels of interleukin-6 was also observed. Moloney et al. (25), applied *Bifidobacterium* to a population experiencing exam stress and found that PSQI scores decreased, indicating improved sleep quality. In Lan et al. (26), study, *Bifidobacterium* was administered to patients with ID, resulting in lower PSQI scores, further demonstrating that *Bifidobacterium* can improve sleep quality.

In the ID group, the PSQI scores were negatively correlated with lTh-FDG and rTh-FDG, indicating that better sleep quality is associated with higher levels of thalamic glucose metabolism. This finding is consistent with the results of (27), whose study demonstrated that glucose metabolism levels in the presumes, posterior cingulate cortex, and thalamus were negatively correlated with PSQI scores. Because the thalamus is involved in regulating the sleep–wake cycle (28), when combined with our finding that thalamic glucose metabolism levels are related to sleep, we inferred that medications such as *Ginkgo biloba* extract, which improves brain glucose metabolism (29), might enhance sleep. A large sample study by Cockle et al. (30), which included 5,028 participants, showed that compared with participants without taking *Ginkgo biloba* extract, participants who had taken *Ginkgo biloba* extract had a significant improvement in sleep quality.

In the ID group, the relative abundance of *Bifidobacterium* was positively correlated with lTh-FDG and rTh-FDG, suggesting that the *Bifidobacterium* supplementation may improve thalamic glucose metabolism levels. In the study by Tong et al. (6), mice fed probiotics containing *Bifidobacterium* demonstrated increased FDG uptake in brain tissue (31). Investigated the mechanism by which probiotics improve brain glucose metabolism in mice and found that brain glucose metabolism was enhanced after oral administration of SLAB51 containing *Bifidobacterium lactis* DSM 32246 (31), noted that glucose transporters (GLUTs) are associated with glucose metabolism, with GLUT3 and GLUT1 being the major neuronal GLUTs. GLUT3 and GLUT1 were significantly increased in treated AD mice compared to untreated AD mice. Adenosine monophosphate-activated protein kinase (AMPK) and protein kinase B (Akt) are key regulators of GLUT expression. Probiotics could reduce the phosphorylation of AMPK and Akt to restore GLUT expression in the brain.

Additionally, the relative abundance of *Roseburia* was positively correlated with lBroca-FDG in the ID group. *Roseburia* is an important intestinal probiotic, belongs to Gram-positive anaerobic bacteria, and is known for producing SCFAs, particularly butyrate (32), conducted a study on 3-year-old Chinese children exposed to polycyclic aromatic hydrocarbons (PAHs) and found that PAHs exposure can lead to a decrease in the relative abundance of *Roseburia* in the children’s gut microbiota, and it may also impair their language abilities. Research by Kort et al. (33) on the language development of 3-year-old rural Ugandan children showed that *Roseburia* was enriched in a population of children with unimpaired language skills, and noted that this was because *Roseburia* is associated with butyrate metabolism.

Regarding FDG-PET, we also found that the glucose metabolism levels of rTh were negatively correlated with those of the lBroca in the non-ID group, but this correlation was not observed in the ID group. Mimura et al. (34), found that when language function was impaired in patients with cerebrovascular disease, the contralateral thalamus would compensate for language function. Integrating these findings with our study, we concluded that when glucose metabolism in lBroca decreases, glucose metabolism in the contralateral thalamus increases to compensate for lBroca’s language function, and ID interferes with this compensatory mechanism.

The correlation among sleep, brain pathology, and the gut microbiota indicates that there may be mutual influences among them. To further explore how gut microbiota and cerebral glucose metabolism are associated with sleep quality in AD patients with ID, we conducted a PLS-SEM analysis and found that *Bifidobacterium* is associated with PSQI through the mediating effect of Th-FDG, rather than through a direct pathway. This finding contributes to understanding the pathophysiological processes of the GBA. Moreover, our study is the first to adopt PLS-SEM in the research of GBA, offering a methodological reference for studies on GBA-related mechanisms and thereby making the research results more comprehensive. Given that the use of hypnotics, especially benzodiazepines, may interfere with the cognitive function of patients with AD, oral administration of probiotics containing *Bifidobacterium* or the application of medications that improve cerebral glucose metabolism may serve as potential alternative treatments for ID in patients with AD (15).

We constructed three models to explore the predictive ability of gut microbiota and brain pathological changes (both individually and in combination) for ID in patients with AD. The results showed that Model 3 outperformed Model 1 and Model 2 in terms of accuracy (0.738), AUC (0.789), AIC (77.95), BIC (84.47), and McFadden’s *R*-squared (0.198). This indicates that considering brain pathological changes and gut microbiota in combination can more effectively explain and predict patients’ ID conditions, providing an important basis for studying the relationships among gut microbiota, brain pathological changes, and ID.

Statistical analyses using relative abundances showed that *Bifidobacterium* exerted a full mediation effect on sleep quality in AD patients with ID via thalamic glucose metabolism (IDE = −0.211, *p* < 0.05). Meanwhile, sensitivity validation did not deviate from this core variable relationship; results demonstrated that after CLR transformation of relative abundances, the mediating effect of Th-FDG remained significant (IDE = −0.227, *p* = 0.008). The two sets of statistical analyses not only showed consistent directions of mediation effects but also both reached statistical significance, indicating that the indirect pathway is not caused by biases in the original data but reflects a genuine biological association. This provides key support for the robustness of the model using relative abundances.

In analyses using relative abundances, *Bifidobacterium* had no direct effect on PSQI (*β* = −0.245, *p* = 0.07). In contrast, sensitivity validation revealed that after CLR transformation, *Bifidobacterium* exerted a significant direct effect on PSQI (*β* = −0.426, *p* = 0.001), with the mediation type shifting from “full mediation” to “partial mediation” (VAF decreasing from 46.37% to 34.76%). This difference arises because CLR transformation eliminates the closure effect of compositional data, uncovering the direct association masked in the original data. Its significance lies in supplementing a “dual-pathway model” for how *Bifidobacterium* influences sleep quality: it acts both through an indirect pathway via thalamic glucose metabolism and through a direct pathway independent of thalamic metabolism (potentially related to *Bifidobacterium* secreting neuroactive substances such as GABA and SCFAs to directly regulate the central nervous system). Although the proportion of the mediation effect decreased, it still exceeded 30%, indicating that the indirect pathway remains an important contributor. The core mechanism is not negated; instead, the mechanistic explanation is rendered more comprehensive.

This research involves neuroscience, microbiology, and imaging. Through these multi-dimensional and comprehensive research approaches, this study is expected to reveal the deep-seated associations that previous studies failed to identify, clarify the roles and interrelationships of various factors in the pathogenesis of AD, provide new targets and strategies for the treatment of sleep disorders in patients with AD, and thus effectively fill the gaps in existing research.

All discussions on mechanisms in this article are drawn from other studies; this study does not delve into in-depth exploration of mechanisms. In the future, we need to further investigate the molecular mechanisms underlying the correlation between gut microbiota and cerebral glucose metabolism in this population. Although this study has shed light on the relationships among brain pathological changes, sleep, and gut microbiota in patients with AD, it still has limitations. In our study, we only excluded patients with special dietary habits (including vegetarianism, ketogenic diet, and intermittent fasting) and those with alcohol use disorder, and did not quantify intake of substances such as caffeine and dietary fiber. Both of these substances may be associated with key study outcomes like gut microbiota and sleep quality; failing to include them in the analysis may have led to the omission of potential confounding factors and also resulted in an incomplete interpretation of the final conclusions. After more than 1 year of follow-up, we found that most patients diagnosed as Aβ-negative in AV45-PET examinations progressed to other types of dementia, such as frontotemporal dementia and Lewy body dementia. Therefore, using Aβ-negative patients as the control group would have compromised the homogeneity of the control group, leading to selection bias; thus, this study did not include an Aβ-negative control group. Furthermore, as our primary objective was to investigate ID specifically in AD populations, and given resource constraints, we prioritized maintaining a robust sample size and research quality within the AD cohort, allowing us to focus on AD-specific factors influencing ID. However, due to the limitations of the cross-sectional design in reliably establishing causal relationships, future research should focus on longitudinal studies. In addition, this study was conducted at a single center. Patients from different regions may vary in genetics and living environments, which might limit the generalizability of the findings. Future longitudinal studies could adopt a multicenter design to minimize selection bias as much as possible.

## 5 Conclusion

In patients with AD, no difference in Aβ deposition was observed between those with normal sleep and those with ID, indicating that Aβ deposition may not be associated with sleep. In AD patients with ID, there may be both a direct and an indirect association between *Bifidobacterium* and sleep quality, with thalamic glucose metabolism mediating the indirect association. This indicates that drugs capable of improving cerebral glucose metabolism or supplementation with probiotics containing *Bifidobacterium* may improve sleep quality. In addition, compensatory interactions may exist between lBroca and the thalamus in terms of glucose metabolism, and this compensation can be disturbed by insomnia.

This study has identified the link through which gut microbiota is associated with sleep quality in AD patients with ID within the GBA. Moreover, it supports the use of drugs that improve cerebral glucose metabolism and probiotic supplements as potential treatments for insomnia in these patients. This study may provide a valuable foundation for future research to better understand the complex sleep mechanisms in this increasingly vulnerable patient population.

## Data Availability

The original contributions presented in the study are included in the article/Supplementary material, further inquiries can be directed to the corresponding authors.

## Glossary

AD: Alzheimer’s disease
AKt: protein kinase B
AMPK: adenosine monophosphate-activated protein kinase
AV45: ^18^F-florbetapir
A*β*: amyloid-β
BMI: body mass index
CT: computed tomography
DSM-5: diagnostic and statistical manual of mental disorders, 5th edition
FDG: ^18^F-fluorodeoxyglucose
FDR: false discovery rate
GABA: gamma-aminobutyric acid
GBA: gut-brain axis
GLUT: glucose transporters
HAM-A: Hamilton anxiety rating scale
HAMD: Hamilton depression rating scale
ID: insomnia disorder
IDE: indirect effect
lAVC: left associative visual cortex
lBroca: left Broca’s area
lCbm: left cerebellum
lCN: left caudate nucleus
LDA: linear discriminant analysis
LEfSe: linear discriminant analysis effect size
lGCa: left anterior cingulate cortex
lGFd: left medial frontal cortex
lGFi: left inferior frontal cortex
lGFm: left middle frontal cortex
lGFs: left superior frontal cortex
liLAT: left inferior lateral anterior temporal cortex
liLPT: left inferior lateral posterior temporal cortex
liPL: left inferior parietal lobule
lLN: left lent form nucleus
lMAT: left anterior medial temporal cortex
lMPT: left posterior medial temporal cortex
lPCC: left posterior cingulate cortex
lPTC: left parietotemporal cortex
lPVC: left primary visual cortex
lsLT: left superior lateral temporal cortex
lSM: left sensorimotor cortex
lsPL: left superior parietal lobule
lTh: left thalamus
MB: midbrain
MMSE: mini-mental state examination
PAHs: polycyclic aromatic hydrocarbons
PCR: polymerase chain reaction
PET: positron emission tomography
PLS-SEM: partial least squares structural equation modeling
PSQI: Pittsburgh sleep quality index
rAVC: right associative visual cortex
rBroca: right Broca’s area
rCbm: right cerebellum
rCN: right caudate nucleus
rGCa: right anterior cingulate cortex
rGFd: right medial frontal cortex
rGFi: right inferior frontal cortex
rGFm: right middle frontal cortex
rGFs: right superior frontal cortex
riLAT: right inferior lateral anterior temporal cortex
riLPT: right inferior lateral posterior temporal cortex
riPL: right inferior parietal lobule
rLN: right lent form nucleus
rMAT: right anterior medial temporal cortex
rMPT: right posterior medial temporal cortex
rPCC: right posterior cingulate cortex
rPTC: right parietotemporal cortex
rPVC: right primary visual cortex
rsLT: right superior lateral temporal cortex
rSM: right sensorimotor cortex
rsPL: right superior parietal lobule
rTh: right thalamus
SCFAs: short-chain fatty acids
SUVRs: standardized uptake value ratios
V: vermis
VAF: variance accounted for
