# Supplementary material for: Study on the relationship between insomnia disorder, PET/CT, and gut microbiota in patients with Alzheimer’s disease
Source: Front Neurol. 2025 Nov 13;16:1669835. doi: 10.3389/fneur.2025.1669835 (PMC12658357; doi:10.3389/fneur.2025.1669835)
Supplement: Supplementary file 1 [file Data_Sheet_1.DOCX]

**SUPPLEMENTARY MATERIAL**

**Supplementary Table 1**1

[**Supplementary Table 2**](#_Toc16134) 2

[**Supplementary Table 3** 4](#_Toc11130)

[**Supplementary Table 4** 5](#_Toc27773)

[**Supplementary Table 5** 5](#_Toc3377)

[**Supplementary Table 6** 5](#_Toc3377)

[**Supplementary Table 7** 6](#_Toc3377)

[**Supplementary Table 8** 6](#_Toc3377)

**Supplementary Table 1.** Comparison of FDG-PET SUVR in each brain region between the ID and Non-ID groups

|  | ID group  (n=30) | Non-ID group  (n=35) | Statistic | p-value | q-value |
| --- | --- | --- | --- | --- | --- |
| rGFs | 1.016±0.058 | 1.000±0.055 | 1.074 | 0.287 | 0.435 |
| lGFs | 0.993±0.048 | 0.968±0.067 | -1.665 | 0.096 | 0.251 |
| rGFd | 1.042±0.063 | 1.044±0.067 | -0.13 | 0.897 | 0.937 |
| lGFd | 1.008±0.057 | 0.986±0.074 | 1.284 | 0.204 | 0.331 |
| rSM | 1.070±0.043 | 1.074±0.035 | -0.396 | 0.694 | 0.768 |
| lSM | 1.085±0.043 | 1.083±0.044 | 0.104 | 0.917 | 0.937 |
| rsPL | 0.972±0.051 | 0.989±0.061 | -1.150 | 0.254 | 0.398 |
| lsPL | 0.962±0.055 | 0.972±0.065 | -0.283 | 0.777 | 0.830 |
| rGFm | 1.104±0.071 | 1.070±0.075 | -1.855 | 0.064 | 0.203 |
| lGFm | 1.099±0.045 | 1.057±0.066 | 3.045 | 0.003 | 0.035* |
| riPL | 1.025±0.056 | 1.041±0.085 | -0.916 | 0.364 | 0.462 |
| liPL | 1.030±0.061 | 1.058±0.084 | -1.543 | 0.128 | 0.286 |
| rGCa | 1.035±0.057 | 1.042±0.059 | -0.592 | 0.554 | 0.651 |
| lGCa | 1.071±0.042 | 1.058±0.072 | 0.872 | 0.387 | 0.472 |
| rAVC | 1.041±0.080 | 1.029±0.071 | -1.698 | 0.090 | 0.249 |
| lAVC | 1.065±0.073 | 1.055±0.074 | -0.855 | 0.392 | 0.472 |
| rBroca | 1.104±0.085 | 1.060±0.080 | 2.144 | 0.036 | 0.169 |
| lBroca | 1.135±0.054 | 1.077±0.080 | 3.402 | 0.001 | 0.024* |
| rPCC | 1.134±0.063 | 1.116±0.065 | 1.174 | 0.116 | 0.273 |
| lPCC | 1.174±0.072 | 1.146±0.071 | -1.816 | 0.069 | 0.203 |
| rPTC | 1.014±0.106 | 0.982±0.078 | -2.132 | 0.033 | 0.169 |
| lPTC | 1.019±0.099 | 1.010±0.071 | 0.426 | 0.671 | 0.768 |
| rsLT | 1.058±0.066 | 1.054±0.068 | -0.066 | 0.948 | 0.948 |
| lsLT | 1.083±0.055 | 1.078±0.055 | 0.383 | 0.703 | 0.768 |
| rCN | 0.993±0.073 | 1.011±0.072 | -1.036 | 0.304 | 0.439 |
| lCN | 0.954±0.093 | 0.968±0.102 | -0.961 | 0.337 | 0.457 |
| rGFi | 1.094±0.081 | 1.069±0.067 | 1.326 | 0.190 | 0.330 |
| lGFi | 1.072±0.057 | 1.046±0.067 | -1.408 | 0.159 | 0.311 |
| rTh | 1.089±0.061 | 1.143±0.067 | -3.027 | 0.002 | 0.031* |
| lTh | 1.069±0.058 | 1.126±0.070 | -3.250 | 0.001 | 0.024* |
| rLN | 1.230±0.075 | 1.298±0.113 | -2.592 | 0.010 | 0.094 |
| lLN | 1.261±0.084 | 1.323±0.124 | -2.158 | 0.031 | 0.170 |
| rPVC | 1.133±0.105 | 1.166±0.090 | -1.354 | 0.181 | 0.331 |
| lPVC | 1.173±0.107 | 1.198±0.109 | -0.938 | 0.352 | 0.456 |
| riLAT | 0.858±0.072 | 0.839±0.064 | -1.500 | 0.134 | 0.286 |
| liLAT | 0.847±0.090 | 0.832±0.064 | -1.290 | 0.197 | 0.331 |
| rMAT | 0.709±0.033 | 0.720±0.051 | -1.028 | 0.308 | 0.438 |
| lMAT | 0.720±0.042 | 0.732±0.053 | -0.961 | 0.340 | 0.457 |
| MB | 0.752±0.037 | 0.781±0.053 | -2.518 | 0.014 | 0.110 |
| riLPT | 0.933±0.068 | 0.902±0.064 | -2.026 | 0.043 | 0.176 |
| liLPT | 0.941±0.060 | 0.920±0.055 | 1.428 | 0.158 | 0.311 |
| rMPT | 0.890±0.034 | 0.902±0.041 | -1.324 | 0.190 | 0.331 |
| lMPT | 0.920±0.038 | 0.937±0.045 | -1.604 | 0.114 | 0.273 |
| Pons | 0.695±0.032 | 0.723±0.053 | -2.000 | 0.045 | 0.176 |
| rCbm | 0.940±0.048 | 0.962±0.049 | -1.868 | 0.066 | 0.203 |
| lCbm | 0.956±0.052 | 0.982±0.048 | -1.954 | 0.051 | 0.184 |
| V | 0.969±0.070 | 1.001±0.061 | -2.125 | 0.034 | 0.170 |

Note: The statistic refers to the t-score (for t-test) or Z-score (for Mann-Whitney U test), depending on the data distribution. ID, insomnia disorder group; rGFs, right superior frontal cortex; lGFs, left superior frontal cortex; rGFd, right medial frontal cortex; lGFd, left medial frontal cortex; rSM, right sensorimotor cortex; lSM, left sensorimotor cortex; rsPL, right superior parietal lobule; lsPL, left superior parietal lobule; rGFm, right middle frontal cortex; lGFm, left middle frontal cortex; riPL, right inferior parietal lobule; liPL, left inferior parietal lobule; rGCa, right anterior cingulate cortex; lGCa, left anterior cingulate cortex; rAVC, right associative visual cortex; lAVC, left associative visual cortex; rBroca, right Broca's area; lBroca, left Broca's area; rPCC, right posterior cingulate cortex; lPCC, left posterior cingulate cortex; rPTC, right parietotemporal cortex; lPTC, left parietotemporal cortex; rsLT, right superior lateral temporal cortex; lsLT, left superior lateral temporal cortex; rCN, right caudate nucleus; lCN, Ieft caudate nucleus; rGFi, right inferior frontal cortex; lGFi, left inferior frontal cortex; rTh, right thalamus; lTh, left thalamus; rLN, right lentiform nucleus; lLN, Ieft lentiform nucleus; rPVC, right primary visual cortex; lPVC, left primary visual cortex; riLAT, right inferior lateral anterior temporal cortex; liLAT, left inferior lateral anterior temporal cortex; rMAT, right anterior medial temporal cortex; lMAT, left anterior medial temporal cortex; MB, midbrain; riLPT, right inferior lateral posterior temporal cortex; liLPT, left inferior lateral posterior temporal cortex; rMPT, right posterior medial temporal cortex; lMPT, left posterior medial temporal cortex; rCbm, right cerebellum; lCbm, left cerebellum; V, vermis; SUVR, standardized uptake value ratio. * q<0.05 indicates a statistically significant difference.

**Supplementary Table 2.** Comparison of AV45-PET SUVR in each brain region between the ID and Non-ID groups

|  | ID group  (n=30) | Non-ID group  (n=35) | Statistic | p-value | q-value |
| --- | --- | --- | --- | --- | --- |
| rGFs | 1.451±0.247 | 1.440±0.309 | 0.158 | 0.875 | 1 |
| lGFs | 1.439±0.261 | 1.431±0.324 | -0.342 | 0.732 | 1 |
| rGFd | 1.269±0.237 | 1.274±0.284 | -0.078 | 0.938 | 1 |
| lGFd | 1.245±0.212 | 1.241±0.287 | -0.217 | 0.828 | 1 |
| rSM | 1.390±0.300 | 1.344±0.238 | -0.651 | 0.515 | 1 |
| lSM | 1.357±0.231 | 1.330±0.266 | -0.546 | 0.585 | 1 |
| rsPL | 1.166±0.224 | 1.154±0.215 | -0.158 | 0.875 | 1 |
| lsPL | 1.228±0.232 | 1.218±0.265 | -0.526 | 0.599 | 1 |
| rGFm | 1.642±0.356 | 1.594±0.307 | -0.553 | 0.580 | 1 |
| lGFm | 1.581±0.398 | 1.540±0.323 | -0.303 | 0.762 | 1 |
| riPL | 1.216±0.201 | 1.170±0.216 | 0.888 | 0.378 | 1 |
| liPL | 1.240±0.246 | 1.212±0.232 | -0.132 | 0.895 | 1 |
| rGCa | 1.360±0.210 | 1.397±0.301 | -0.575 | 0.567 | 1 |
| lGCa | 1.359±0.245 | 1.429±0.321 | -0.915 | 0.360 | 1 |
| rAVC | 1.600±0.462 | 1.538±0.245 | -0.039 | 0.969 | 1 |
| lAVC | 1.637±0.472 | 1.601±0.276 | -0.382 | 0.703 | 1 |
| rBroca | 1.567±0.417 | 1.477±0.313 | -0.579 | 0.563 | 1 |
| lBroca | 1.465±0.382 | 1.378±0.294 | -1.026 | 0.305 | 1 |
| rPCC | 1.501±0.269 | 1.543±0.311 | -0.882 | 0.378 | 1 |
| lPCC | 1.514±0.293 | 1.528±0.310 | -0.447 | 0.655 | 1 |
| rPTC | 1.449±0.334 | 1.417±0.283 | -0.566 | 0.572 | 1 |
| lPTC | 1.543±0.427 | 1.452±0.276 | -1.066 | 0.286 | 1 |
| rsLT | 1.409±0.367 | 1.392±0.257 | -0.487 | 0.626 | 1 |
| lsLT | 1.397±0.415 | 1.355±0.274 | -0.250 | 0.803 | 1 |
| rCN | 1.015±0.326 | 1.062±0.338 | -0.569 | 0.572 | 1 |
| lCN | 1.064±0.318 | 1.136±0.359 | -0.849 | 0.399 | 1 |
| rGFi | 1.367±0.391 | 1.299±0.311 | -0.434 | 0.664 | 1 |
| lGFi | 1.390±0.456 | 1.306±0.334 | -0.500 | 0.617 | 1 |
| rTh | 1.216±0.255 | 1.292±0.197 | -1.171 | 0.242 | 1 |
| lTh | 1.203±0.243 | 1.294±0.197 | -1.619 | 0.106 | 1 |
| rLN | 1.432±0.197 | 1.492±0.249 | -1.250 | 0.211 | 1 |
| lLN | 1.414±0.176 | 1.498±0.277 | -1.500 | 0.134 | 1 |
| rPVC | 1.328±0.276 | 1.298±0.302 | -0.355 | 0.722 | 1 |
| lPVC | 1.303±0.353 | 1.302±0.267 | -0.474 | 0.636 | 1 |
| riLAT | 1.416±0.350 | 1.364±0.238 | -0.566 | 0.572 | 1 |
| liLAT | 1.387±0.384 | 1.355±0.268 | -0.158 | 0.875 | 1 |
| rMAT | 0.930±0.124 | 0.949±0.187 | 0.000 | 1.000 | 1 |
| lMAT | 0.901±0.120 | 0.927±0.153 | -0.053 | 0.958 | 1 |
| MB | 1.536±0.253 | 1.485±0.227 | 0.842 | 0.403 | 1 |
| riLPT | 1.490±0.416 | 1.409±0.299 | -0.54 | 0.590 | 1 |
| liLPT | 1.403±0.585 | 1.353±0.350 | -0.211 | 0.833 | 1 |
| rMPT | 1.122±0.116 | 1.136±0.152 | -0.424 | 0.673 | 1 |
| lMPT | 1.060±0.137 | 1.072±0.152 | -0.026 | 0.979 | 1 |
| Pons | 1.467±0.191 | 1.426±0.249 | 0.739 | 0.463 | 1 |
| rCbm | 0.983±0.031 | 0.980±0.016 | -1.204 | 0.229 | 1 |
| lCbm | 1.017±0.031 | 1.020±0.016 | -1.204 | 0.229 | 1 |
| V | 1.022±0.153 | 1.035±0.139 | -0.353 | 0.725 | 1 |

Note: The statistic refers to the t-score (for t-test) or Z-score (for Mann-Whitney U test), depending on the data distribution. ID, insomnia disorder group; rGFs, right superior frontal cortex; lGFs, left superior frontal cortex; rGFd, right medial frontal cortex; lGFd, left medial frontal cortex; rSM, right sensorimotor cortex; lSM, left sensorimotor cortex; rsPL, right superior parietal lobule; lsPL, left superior parietal lobule; rGFm, right middle frontal cortex; lGFm, left middle frontal cortex; riPL, right inferior parietal lobule; liPL, left inferior parietal lobule; rGCa, right anterior cingulate cortex; lGCa, left anterior cingulate cortex; rAVC, right associative visual cortex; lAVC, left associative visual cortex; rBroca, right Broca's area; lBroca, left Broca's area; rPCC, right posterior cingulate cortex; lPCC, left posterior cingulate cortex; rPTC, right parietotemporal cortex; lPTC, left parietotemporal cortex; rsLT, right superior lateral temporal cortex; lsLT, left superior lateral temporal cortex; rCN, right caudate nucleus; lCN, Ieft caudate nucleus; rGFi, right inferior frontal cortex; lGFi, left inferior frontal cortex; rTh, right thalamus; lTh, left thalamus; rLN, right lentiform nucleus; lLN, Ieft lentiform nucleus; rPVC, right primary visual cortex; lPVC, left primary visual cortex; riLAT, right inferior lateral anterior temporal cortex; liLAT, left inferior lateral anterior temporal cortex; rMAT, right anterior medial temporal cortex; lMAT, left anterior medial temporal cortex; MB, midbrain; riLPT, right inferior lateral posterior temporal cortex; liLPT, left inferior lateral posterior temporal cortex; rMPT, right posterior medial temporal cortex; lMPT, left posterior medial temporal cortex; rCbm, right cerebellum; lCbm, left cerebellum; V, vermis; SUVR, standardized uptake value ratio. All q-values are greater than 0.05, indicating no statistically significant differences.

**Supplementary Table 3.** Multiple correlation analysis of the ID group

|  |  | r | p-value | q-value |
| --- | --- | --- | --- | --- |
| Bifidobacterium | lBroca-FDG | -0.278 | 0.136 | 0.283 |
|  | lTh-FDG | 0.519 | 0.003 | 0.013* |
|  | rTh-FDG | 0.509 | 0.004 | 0.014* |
|  | lGFm-FDG | -0.240 | 0.201 | 0.387 |
|  | PSQI | -0.637 | <0.001 | <0.001** |
| Prevotella_7 | lBroca-FDG | 0.119 | 0.531 | 0.742 |
|  | lTh-FDG | -0.345 | 0.0621 | 0.155 |
|  | rTh-FDG | -0.193 | 0.307 | 0.512 |
|  | lGFm-FDG | 0.024 | 0.899 | 0.977 |
|  | PSQI | 0.369 | 0.045 | 0.125 |
| Roseburia | lBroca-FDG | 0.449 | 0.013 | 0.041* |
|  | lTh-FDG | 0.230 | 0.222 | 0.396 |
|  | rTh-FDG | 0.159 | 0.401 | 0.627 |
|  | lGFm-FDG | 0.299 | 0.108 | 0.246 |
|  | PSQI | -0.029 | 0.877 | 0.977 |
| lBroca-FDG | lGFm-FDG | 0.796 | <0.001 | <0.001** |
|  | lTh-FDG | -0.013 | 0.945 | 0.984 |
|  | rTh-FDG | -0.09 | 0.636 | 0.795 |
|  | PSQI | 0.04 | 0.833 | 0.977 |
| lGFm-FDG | lTh-FDG | -0.003 | 0.988 | 0.988 |
|  | rTh-FDG | -0.095 | 0.616 | 0.795 |
|  | PSQI | 0.118 | 0.534 | 0.742 |
| lTh-FDG | rTh-FDG | 0.818 | <0.001 | <0.001** |
|  | PSQI | -0.585 | 0.001 | 0.005** |
| rTh-FDG | PSQI | -0.612 | <0.001 | <0.001** |

FDG, ^18^F-fluorodeoxyglucose positron emission tomography; lGFm, left middle frontal cortex; lBroca, left Broca's area; lTh, left thalamus; rTh, right thalamus; r, correlation coefficient. *q<0.05 indicates a significant correlation. **q< 0.01 indicates a highly significant correlation.

**Supplementary Table 4.** Multiple correlation analysis of the Non-ID group

|  |  | r | p-value | q-value |
| --- | --- | --- | --- | --- |
| Bifidobacterium | lBroca-FDG | 0.153 | 0.069 | 0.133 |
|  | lTh-FDG | -0.125 | 0.952 | 0.952 |
|  | rTh-FDG | -0.108 | 0.899 | 0.952 |
|  | lGFm-FDG | 0.0415 | 0.32 | 0.444 |
|  | PSQI | -0.081 | 0.933 | 0.952 |
| Prevotella_7 | lBroca-FDG | 0.368 | 0.009 | 0.050 |
|  | lTh-FDG | 0.481 | 0.025 | 0.069 |
|  | rTh-FDG | 0.300 | 0.054 | 0.113 |
|  | lGFm-FDG | 0.216 | 0.031 | 0.078 |
|  | PSQI | 0.009 | 0.477 | 0.627 |
| Roseburia | lBroca-FDG | 0.251 | 0.01 | 0.050 |
|  | lTh-FDG | 0.305 | 0.013 | 0.054 |
|  | rTh-FDG | 0.198 | 0.047 | 0.107 |
|  | lGFm-FDG | 0.079 | 0.172 | 0.307 |
|  | PSQI | -0.037 | 0.692 | 0.865 |
| lBroca-FDG | lGFm-FDG | 0.859 | <0.001 | <0.001** |
|  | lTh-FDG | -0.401 | 0.017 | 0.061 |
|  | rTh-FDG | -0.528 | 0.001 | 0.008** |
|  | PSQI | -0.214 | 0.217 | 0.339 |
| lGFm-FDG | lTh-FDG | -0.228 | 0.188 | 0.313 |
|  | rTh-FDG | -0.391 | 0.020 | 0.063 |
|  | PSQI | -0.188 | 0.280 | 0.412 |
| lTh-FDG | rTh-FDG | 0.897 | <0.001 | <0.001** |
|  | PSQI | -0.056 | 0.748 | 0.890 |
| rTh-FDG | PSQI | -0.047 | 0.787 | 0.894 |

FDG, ^18^F-fluorodeoxyglucose positron emission tomography; lGFm, left middle frontal cortex; lBroca, left Broca's area; lTh, left thalamus; rTh, right thalamus; r, correlation coefficient. **q<0.01 indicates a highly significant correlation.

**Supplementary Table 5.** The PLS-SEM structural model with Th-FDG as the mediating variable

| Path | Original Sample | SD | t | p-value |
| --- | --- | --- | --- | --- |
| Total path: Bifidobacterium→PSQI | -0.455 | 0.106 | 4.278 | <0.001** |
| Indirect Path: Bifidobacterium→FDG→PSQI | -0.211 | 0.103 | 2.037 | 0.042* |
| Direct Path: Bifidobacterium→PSQI | -0.245 | 0.136 | 1.804 | 0.071 |
| Direct Path: Bifidobacterium→FDG | 0.354 | 0.161 | 2.194 | 0.028* |
| Direct Path: FDG→PSQI | -0.595 | 0.121 | 4.928 | <0.001** |

"Original sample" in the table indicates the raw values of the path coefficient, indirect effect, or total effect.

**Supplementary Table 6.** Reliability and validity indicators of the PLS-SEM measurement model with Th-FDG as the mediating variable

| Latent Variable | Observed Variable | Factor Loading | CR | Cronbach's α | AVE | 95% Confidence Interval of HTMT |
| --- | --- | --- | --- | --- | --- | --- |
| FDG | lTh-FDG | 0.971 | 0.970 | 0.939 | 0.943 | Does not include 1 |
|  | rTh-FDG | 0.970 |  |  |  |  |

**Supplementary Table 7.** The PLS-SEM structural model with Bifidobacterium as the mediating variable

| Path | Original Sample | SD | t | p-value |
| --- | --- | --- | --- | --- |
| Total path: Bifidobacterium→PSQI | 0.354 | 0.161 | 2.194 | 0.028* |
| Indirect Path: Bifidobacterium→  FDG→PSQI | -0.087 | 0.058 | 1.494 | 0.135 |
| Direct Path: Bifidobacterium→PSQI | -0.245 | 0.136 | 1.804 | 0.071 |
| Direct Path:  FDG→Bifidobacterium | 0.354 | 0.161 | 2.194 | 0.028* |
| Direct Path: FDG→PSQI | -0.595 | 0.121 | 4.928 | <0.001** |

"Original sample" in the table indicates the raw values of the path coefficient, indirect effect, or total effect.

**Supplementary Table 8.** Reliability and validity indicators of the PLS-SEM measurement model with Bifidobacterium as the mediating variable

| Latent Variable | Observed Variable | Factor Loading | CR | Cronbach's α | AVE | 95% Confidence Interval of HTMT |
| --- | --- | --- | --- | --- | --- | --- |
| Th-FDG | lTh-FDG | 0.971  0.970 | 0.970 | 0.939 | 0.943 | Does not include 1 |
|  | rTh-FDG |  |  |  |  |  |
